# Supplementary material for: NanoPhotoNet-Lase: Physics-Informed Neural Networks for Accelerated Design of Coherent Metasurface Nanolasers
Source: Nanomaterials (Basel). 2026 Sep 22;16(19):1200. doi: 10.3390/nano16191200 (PMC13667036; doi:10.3390/nano16191200)
Supplement: Supplementary file 1 [file nanomaterials-16-01200-s001.zip › nanomaterials-4574752-supplementary.pdf]

Supporting Information:

NanoPhotoNet-Lase: Physics-Informed Neural Networks for Accelerated Design of Coherent Metasurface Nanolasers

Omar A. M. Abdelraouf [1, 2]

Affiliations:

[1] Engineering Physics and Mathematics Department, Faculty of Engineering, Ain Shams University, Cairo 11535, Egypt

[2] Institute of Materials Research and Engineering, Agency for Science, Technology, and Research (A*STAR), 2 Fusionopolis Way, #08-03, Innovis, Singapore 138634, Singapore

Corresponding author: Omar A.M. Abdelraouf (email: omar.abdelraouf@eng.asu.edu.eg)

Table of Contents:

S1. Theoretical framework of PINNs nanolaser

S2. NanoPhotoNet-Lase architecture and training

S3. Measured refractive indices used in PINNs nanolaser metasurfaces

S4. NanoPhotoNet-Lase: Single resonance inverse design

S5. NanoPhotoNet-Lase: Dual resonance inverse design

S6. Fabrication flow of PINNs nanolaser metasurface

S7. Nanolasers optical measurement setup

S8. Phase versus PINNs nanolaser meta-atom rotation

S9. PINNs nanolaser metalens

S10. PINNs nanolaser beam steering

S11. Benchmark with nanolasers metasurfaces

S1. Theoretical framework of nanolaser physics

The realization of coherent metasurface nanolasers requires the simultaneous treatment of two strongly coupled physical domains: the electromagnetic response of subwavelength resonant nanostructures and the nonequilibrium carrier dynamics of the active gain medium. In conventional numerical frameworks, these processes are solved independently through iterative coupling between Maxwell solvers and rate-equation formalisms, resulting in substantial computational complexity and long simulation times. In NanoPhotoNet-Lase, these physical processes are embedded directly into a unified physics-informed neural network (PINN) framework, enabling self-consistent prediction of optical fields, polarization dynamics, and lasing thresholds from arbitrary metasurface geometries and material distributions. The theoretical formulation underlying NanoPhotoNet-Lase is based on the coupled solution of Maxwell's equations, Lorentz polarization dynamics, and four-level carrier rate equations. These equations collectively define the physical constraints incorporated into the network loss function during training and iterative forward design optimization.

Electromagnetic Formulation and Vector Helmholtz Equation

The passive optical response of the metasurface resonator is governed by the frequency-domain Maxwell equations. For time-harmonic fields with angular frequency (ω), the electromagnetic modes supported by the nanostructure satisfy the vector Helmholtz equation:

$$\nabla \times \nabla \times E(r) - k_0^2 \varepsilon_r(r) E(r) = 0 \quad \text{(S1)}$$

where $E(r)$ denotes the electric field vector, ($k_0 = \frac{\omega}{c}$) is the free-space wavenumber, and $\varepsilon_r(r)$ represents the spatially varying dielectric permittivity determined by the geometry and material composition of the metasurface.

Within NanoPhotoNet-Lase, the electromagnetic field is not computed using conventional spatial discretization schemes such as finite-difference time-domain (FDTD) or finite-element methods (FEM). Instead, PINN learns field solutions by minimizing the residuals of Maxwell's equations over randomly sampled collocation points distributed throughout the computational

domain. The domain includes the dielectric nanopillars, substrate, surrounding medium, and gain layer. The dielectric function $\varepsilon_r(r)$ is parameterized directly through the geometrical descriptors of the metasurface, including trapezoidal dimensions (L_1, L_2, L, D), height (h), and lattice periodicity (p).

For periodic metasurface arrays, Bloch periodic boundary conditions are imposed along the in-plane directions. The electromagnetic field therefore satisfies Bloch's theorem:

$$E(r + R) = E(r)e^{ik \cdot R} \quad \text{(S2)}$$

Where R is the lattice translation vector and k is the Bloch wavevector associated with the incident excitation. Along the out-of-plane direction, absorbing boundary conditions are required to emulate open radiative systems and suppress artificial reflections. These conditions are implicitly learned within the PINN framework through physics residual minimization and field continuity constraints.

Four-Level Gain Dynamics and Population Inversion

To describe the active lasing state, NanoPhotoNet-Lase incorporates a four-level laser model appropriate for organic dye systems such as Rhodamine B (Figure S1). The gain medium consists of four energy states corresponding to the ground state, lower laser level, upper laser level, and pump level. The total molecular density satisfies the conservation condition:

$$N_t = N_0 + N_1 + N_2 + N_3 \quad \text{(S3)}$$

Where N_i denotes the population density of the (i)-th energy level.

The temporal evolution of the carrier populations is governed by coupled rate equations. The pump level population N_3 evolves according to:

$$\frac{dN_3}{dt} = W_p N_0 - \frac{N_3}{\tau_{32}} - \frac{1}{\hbar\omega_p} E_p \cdot \frac{\partial P_p}{\partial t} \quad \text{(S4)}$$

Where W_p is the optical pumping rate, τ_{32} is the nonradiative relaxation lifetime from the pump level to the upper laser level, and P_p denotes the polarization associated with the pump transition.

The upper laser level population N_2 satisfies:

$$\frac{dN_2}{dt} = \frac{N_3}{\tau_{32}} - \frac{N_2}{\tau_{21}} - \frac{1}{\hbar\omega_l} E_l \cdot \frac{\partial \boldsymbol{P}_l}{\partial t} \tag{S5}$$

where τ_{21} corresponds to the spontaneous emission lifetime and P_l is the lasing-transition polarization density.

Similarly, the lower laser level population evolves as:

$$\frac{dN_1}{dt} = \frac{N_2}{\tau_{21}} - \frac{N_1}{\tau_{10}} + \frac{1}{\hbar\omega_l} E_l \cdot \frac{\partial \boldsymbol{P}_l}{\partial t} \tag{S6}$$

Efficient lasing requires rapid depopulation of the lower laser level ($\tau_{10} \ll \tau_{21}$) to sustain population inversion:

$$\Delta N = N_2 - N_1 > 0 \tag{S7}$$

The optical gain coefficient can then be expressed as:

$$g = \sigma_e(N_2 - N_1) \tag{S8}$$

where σ_e is the stimulated emission cross-section of the gain medium.

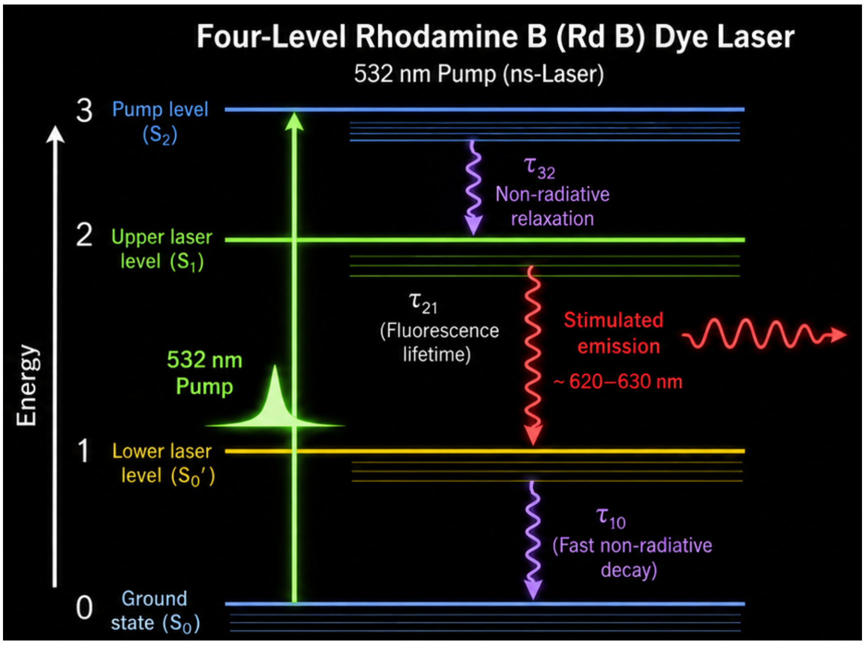

Figure S1. Carrier dynamics in Rd B upon ns-laser pump.

Lorentz Polarization Dynamics and Light–Matter Interaction

The interaction between the electromagnetic field and molecular transitions is mediated through the macroscopic polarization density. The polarization response is modeled using the Lorentz oscillator formalism:

$$\frac{d^2\boldsymbol{P}}{dt^2} + \gamma \frac{d\boldsymbol{P}}{dt} + \omega_0^2 P = \kappa \Delta N E \tag{S9}$$

where γ denotes the dephasing rate, ω_0 is the transition resonance frequency, and κ is the light–matter coupling coefficient related to the dipole transition moment μ:

$$\kappa = \frac{2\omega_0 |\mu|^2}{3\hbar} \tag{S10}$$

This formulation establishes a self-consistent feedback loop between optical confinement and carrier dynamics. The electric field drives polarization oscillations, polarization modifies population inversion, and the resulting gain or absorption subsequently alters the electromagnetic field distribution. NanoPhotoNet-Lase is therefore trained to identify fixed-point solutions of this nonlinear coupled system.

Bound States in the Continuum and Quasi-BIC Nanolasing

The exceptionally low lasing thresholds achieved in metasurface nanolasers arise from bound states in the continuum (BICs). BICs are non-radiative eigenmodes embedded within the continuum of radiative states yet remain spatially localized due to symmetry protection or destructive interference. In the ideal limit, BIC modes exhibit theoretically infinite quality factors: $Q \rightarrow \infty$. However, perfectly confined modes cannot couple efficiently to free-space radiation and therefore cannot emit useful laser output. Practical metasurface nanolasers therefore operate in the quasi-BIC regime, where slight symmetry perturbations intentionally introduce finite radiative leakage while preserving high optical confinement. In Nb_2O_5 metasurfaces, quasi-BIC resonances emerge through diffractive coupling between electric dipole and magnetic dipole Mie resonances. The resulting super cavity modes exhibit strong near-field localization and enhanced overlap with the Rhodamine B gain medium, thereby reducing lasing threshold and enabling coherent directional emission

S2. NanoPhotoNet-Lase architecture and training

NanoPhotoNet-Lase is implemented as a physics-informed neural network designed for accelerated forward design of coherent metasurface nanolasers. Unlike conventional data-driven neural networks that rely solely on labeled simulation datasets, the proposed framework embeds the governing physical equations directly into the optimization process, enabling physically interpretable predictions with significantly reduced training data requirements.

The network architecture consists of spatial point clouds describing arbitrary metasurface geometries. Each point within the computational domain is represented by a feature vector containing spatial coordinates and local refractive index information:

$$x_i = (x_i, y_i, z_i, n_i) \tag{S11}$$

The point-cloud representation allows NanoPhotoNet-Lase to naturally handle geometrically irregular and sparse nanophotonic structures without requiring voxelization or structured meshing.

Shared multilayer perceptrons (MLPs) are first applied independently to each point to extract local geometric and material features. A symmetric max-pooling operation subsequently aggregates global structural information into a latent descriptor vector that remains invariant to point ordering. This global feature representation encodes collective metasurface properties such as periodicity, refractive-index contrast, and resonant coupling strength. Local and global features are then fused to predict the complex-valued electromagnetic field and polarization distributions throughout the structure.

The network outputs include the real and imaginary components of the electric field:

$$E(r) = E_r(r) + iE_i(r) \tag{S12}$$

as well as the corresponding polarization densities and carrier population distributions.

Physics-Informed Loss Functions

The total loss function consists of a weighted combination of electromagnetic physics residuals, carrier dynamics constraints, lasing conditions, and sparse supervised field data:

$$\mathcal{L}_{total} = \lambda_{EM}\mathcal{L}_{EM} + \lambda_{rate}\mathcal{L}_{rate} + \lambda_{gain}\mathcal{L}_{gain} + \lambda_{data}\mathcal{L}_{data} \quad \text{(S13)}$$

The electromagnetic residual loss enforces Maxwell's equations throughout the computational domain:

$$\mathcal{L}_{EM} = \frac{1}{N_c}\sum_{i=1}^{Nc}|\nabla \times \nabla \times \boldsymbol{E_i} - k_0^2\varepsilon_r\boldsymbol{E_i}|^2 \quad \text{(S14)}$$

where N_c denotes the number of collocation points. Spatial derivatives are computed through automatic differentiation.

The carrier-dynamics loss enforces the steady-state rate equations and Lorentz polarization dynamics:

$$\mathcal{L}_{rate} = \sum_j \left|\frac{dN_j}{dt}\right|^2 + \left|\frac{d^2\boldsymbol{P}}{dt^2} + \gamma\frac{d\boldsymbol{P}}{dt} + \omega_0^2\boldsymbol{P} - \kappa\Delta N\boldsymbol{E}\right|^2 \quad \text{(S15)}$$

To explicitly guide the network toward lasing solutions, a gain-threshold loss is introduced:

$$\mathcal{L}_{gain} = max(0, \alpha_{loss} - \Gamma g) \quad \text{(S16)}$$

where Γ is the optical confinement factor and α_{loss} represents cavity losses derived from the resonance quality factor.

Although NanoPhotoNet-Lase primarily relies on physics-informed learning, sparse reference electromagnetic fields obtained from FDTD simulations are incorporated to stabilize optimization and prevent convergence toward trivial field solutions:

$$\mathcal{L}_{data} = \frac{1}{N_r}\sum_{i=1}^{N_r}|\boldsymbol{E_{PINN}}(\boldsymbol{r_i}) - \boldsymbol{E}_{FDTD}(\boldsymbol{r_i})|^2 \quad \text{(S17)}$$

where N_r is the number of reference sampling points.

Iterative Forward Design Model Training, Speed, and Optimization

NanoPhotoNet-Lase takes a 4-dimensional input (spatial coordinates and local refractive index) and predicts an 86×33×51 3D field tensor. The encoder comprises a 3-layer MLP with 256, 512, and 128 hidden neurons, producing a latent descriptor that is reshaped into a base spatial tensor. The decoder consists of three 3D transposed-convolution layers (64, 32, and 16 channels),

an intermediate 64-channel 3D residual block, and a final 1-channel 3D convolution, followed by trilinear interpolation to match the target output dimensions. ReLU activation functions are used across all intermediate layers. Collocation points were sampled using a PointNet-based strategy, with denser sampling at smaller mesh sizes to resolve fine geometric features critical to quasi-BIC excitation.

Training data spanned multiple metasurface designs with scaled dimensions to cover a broad range of high-Q resonance wavelengths, with dense sampling around each resonance peak to maximize spectral and field-prediction resolution. Network depth, activation functions, and learning rate were optimized to enhance prediction accuracy and convergence stability. Convergence was verified across varied initial geometries and gain parameters, consistently reaching the same solution branch, though prediction accuracy degrades gradually as Q approaches the ideal lossless limit.

All training and inference were performed on a workstation with an NVIDIA GPU (8 GB GPU RAM) and 256 GB system RAM. FDTD simulations used a fixed mesh size of 10 nm in-plane and 5 nm out-of-plane, with a typical runtime of 200~300 sec per design. The training dataset consisted of 725 FDTD simulations, requiring approximately 60 hours of total generation time, a one-time cost amortized once the model is trained. Subsequent forward predictions and inverse-design iterations benefit from the full per-evaluation speedup, though this does not include the upfront dataset-generation and training cost. A single forward pass of NanoPhotoNet-Lase requires <1 msec, corresponding to an acceleration of $\sim 10^5$ over FDTD for forward prediction. Inverse design applies this forward model iteratively (in the order of hundreds of iterations, completing within a few seconds), and while this remains substantially faster than iterative FDTD-based optimization, the stated $\times 10^5$ acceleration factor strictly refers to single forward-evaluation speed rather than total inverse-design runtime.

After training, NanoPhotoNet-Lase operates as an accelerated forward-design engine for coherent metasurface nanolasers. Given arbitrary geometric parameters, material indices, and gain conditions, the network predicts the corresponding electromagnetic field distribution, quality factor, modal confinement, population inversion, and lasing threshold within milliseconds. An iterative optimization workflow is subsequently employed to converge toward target lasing specifications. Geometrical parameters such as nanopillar length, width, height, periodicity, and

refractive index are continuously updated while the trained PINN rapidly evaluates the resulting optical response. The iterative process continues until the predicted design simultaneously satisfies the desired emission wavelength, lasing threshold, quality factor, and beam-shaping characteristics.

This hybrid strategy combining physics-informed learning, sparse electromagnetic supervision, and iterative forward optimization enables NanoPhotoNet-Lase to bypass the computational bottlenecks of conventional full-wave simulations while preserving rigorous physical consistency. Consequently, the framework establishes a scalable pathway toward real-time design of coherent active metasurfaces, programmable nanolaser arrays, and AI-assisted nonlinear photonic systems.

S3. Measured refractive indices used in PINNs nanolaser metasurfaces

Figure S2 shows the refractive indices of Nb_2O_5 used materials in fabricating the PINNs nanolaser metasurfaces, measured by variable angle ellipsometry (VASE).

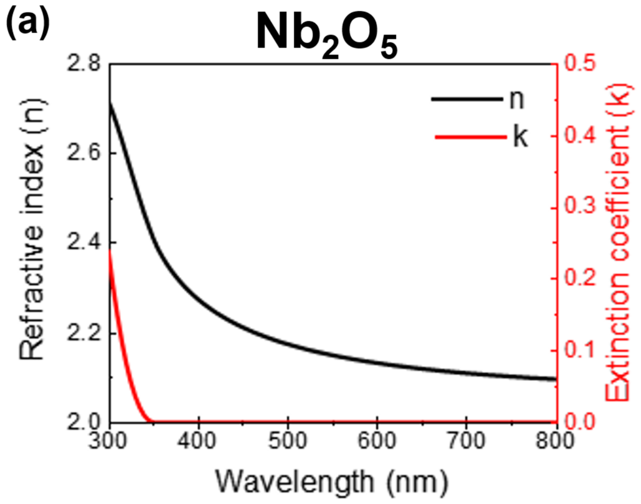

Figure S2. Measured refractive index of materials in the tunable BIC metasurface. (a) amorphous niobium pentoxide (Nb_2O_5).

S4. NanoPhotoNet-Lase: Single resonance inverse design

Figure S3 demonstrates the performance of the NanoPhotoNet-Lase framework in the inverse design of a single resonant reflection spectrum. To enable on-demand nanolasing at arbitrary wavelengths, the model was tasked with predicting structural parameters that yield a resonance with a similar *Q*-factor across a broadband wavelength range of 620 nm to 700 nm. The AI-predicted reflection spectra perfectly reconstruct the desired reflection response with high efficiency. These precise spectral matching underscores the advantage of embedding the physics of the electric field to accurately inverse-design nanoscale light–matter interactions.

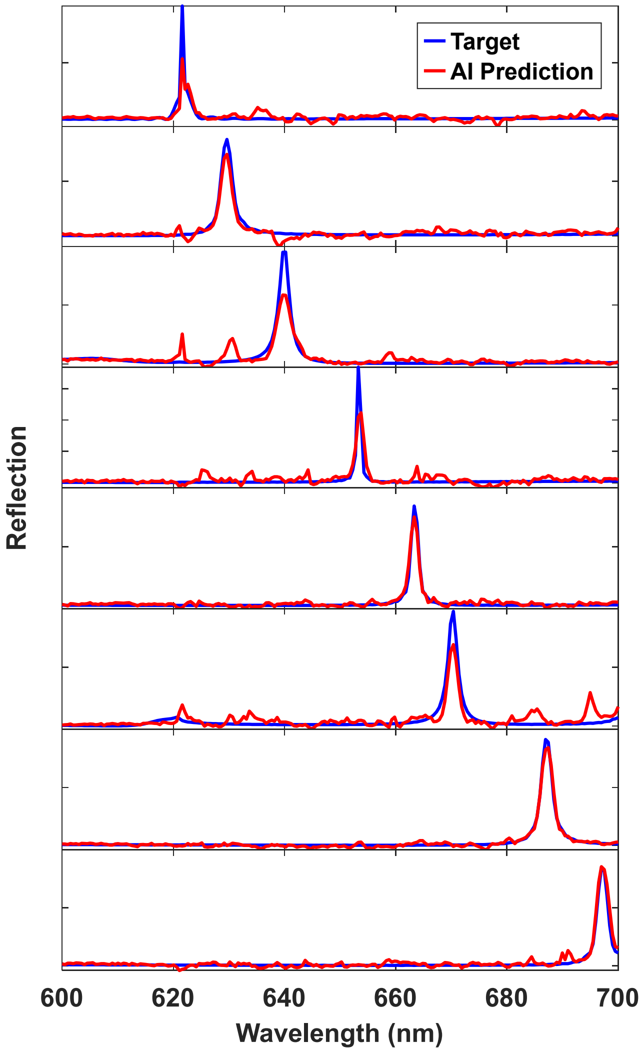

Figure S3. Target resonant reflection response across a broadband wavelength range and the corresponding AI-predicted response for single-resonance MLMs hybrid nanostructures.

S5. NanoPhotoNet-Lase: Dual resonance inverse design

The NanoPhotoNet-Lase framework was further evaluated for the inverse design of high-Q dual-resonant reflection spectra. This dual-resonance profile is critical for facilitating strong light coupling at both the pump and emission wavelengths. As shown in Figure S4, the predicted dual resonances exhibit a tunable wavelength separation between pump and emission peaks ranging from 20 nm to 65 nm. This adaptability accommodates large gain materials with diverse emission peaks, enabling the realization of on demand nanolasers at arbitrary wavelengths. The AI-predicted reflection profile perfectly reconstructs the target response with high efficiency. Ultimately, this highlights the robustness of utilizing electric field physics to accurately inverse-design complex light–matter interactions within dual-resonant cavities at the nanoscale.

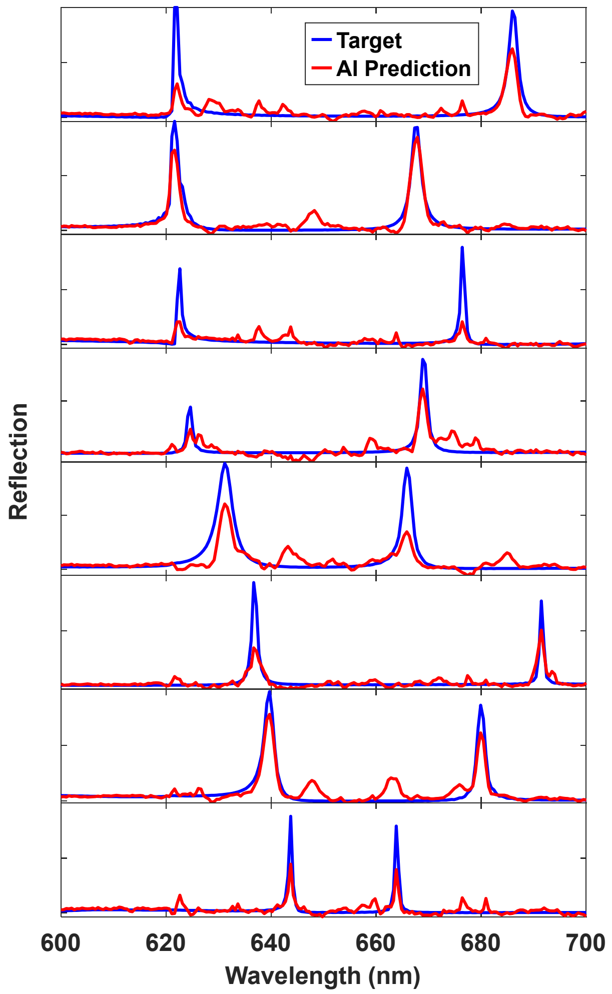

Figure S4. Target dual-resonant reflection response across a broadband wavelength range and the corresponding AI-predicted response for dual-resonant MLMs hybrid nanostructures.

S6. Fabrication flow of PINNs nanolaser metasurface

Figure S5 shows the fabrication flow of PINNs nanolaser metasurface in the visible using an Nb_2O_5 BIC metasurface.

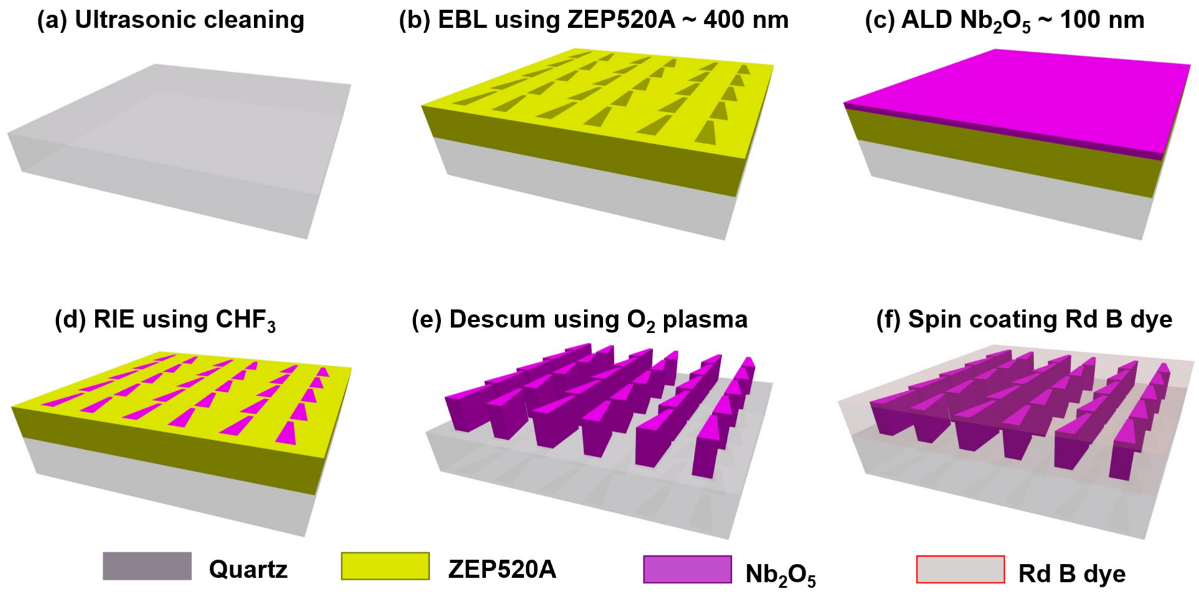

Figure S5. Fabrication flow of the PINNs nanolaser Nb_2O_5 BIC metasurface.

S7. Nanolasers optical measurement setup

The optical setup used in all nanolaser measurements of predicted resonant Nb2O5 BIC metasurface cavities is shown in Figure S6. The detector, spectrometer, and a fiber-coupled nanosecond ns-laser at a wavelength of 532 nm were used and spectrometer from a WITec system with an laser repetition rate 10 Hz and average power of up to 1,500 nW.

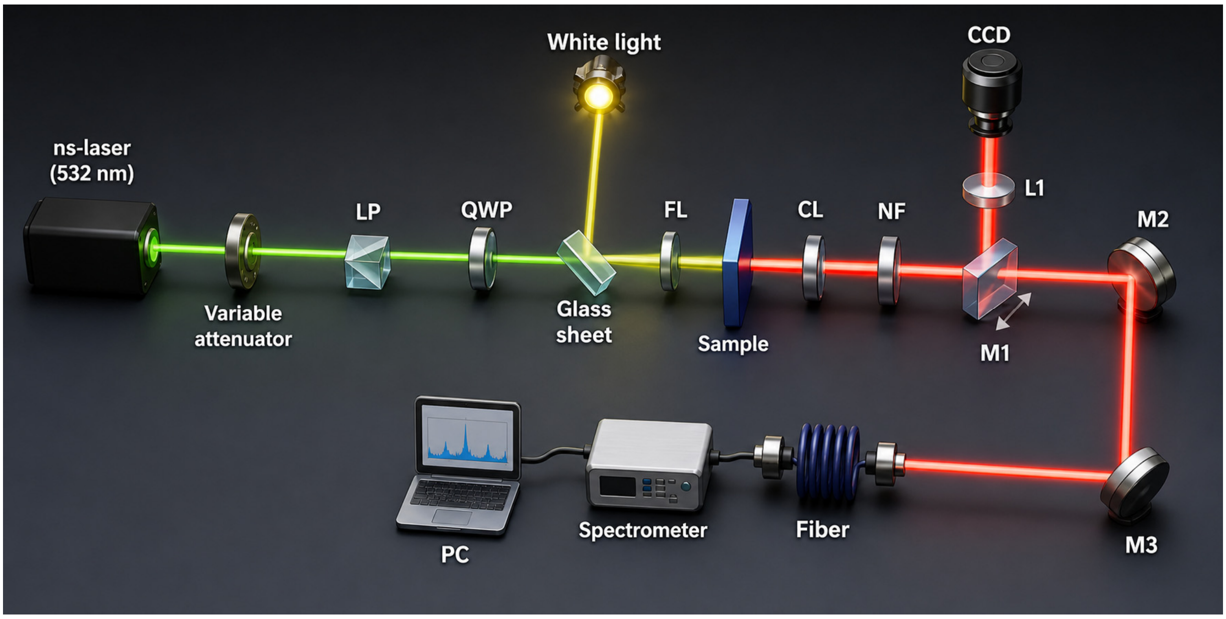

Figure S6. Optical setup used for nanolaser measurements.

S8. Phase versus PINNs nanolaser meta-atom rotation

Figure S7 illustrates the relative transmission magnitude and spatial phase response of BIC meta-atoms, which were inversely designed using the NanoPhotoNet-Lase framework. To enable precise continuous wavefront modulation near the resonant lasing emission wavelength of 620 nm, the phase gradient was engineered by leveraging the Pancharatnam-Berry (PB) phase concept. By systematically varying the in-plane rotation angle of the trapezoidal pillars with respect to the *z*-axis, the AI-optimized structures achieve a complete 2π phase span. Crucially, this robust phase control is accompanied by an exceptional transmission efficiency approaching 98% at the target wavelength.

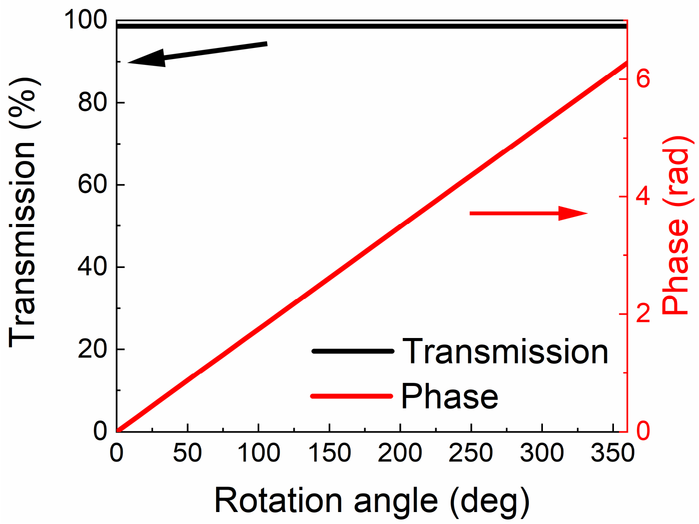

Figure S7. Relative transmission magnitude and PB phase response of the NanoPhotoNet-Lase inversely designed BIC meta-atom near the 620 nm resonant wavelength vs rotation angle.

S9. PINNs nanolaser metalens

The nanolaser metalens was designed and simulated by first arranging the meta-atoms across the metasurface to accurately tailor the far-field scattering profile, thereby directing the emitted light toward a focal point located at the prescribed focal length of the lens. The phase distribution of the metalens followed an ideal hyperbolic phase profile, which can be expressed as::[1]

$$\varphi(x,y) = \frac{2\pi}{\lambda}\left(f - \sqrt{f^2 + x^2 + y^2} - f\right) \tag{S18}$$

where f = 3.5 um represents the focal length, $\boldsymbol{\lambda}$ = 620 nm is the operating wavelength, and the metalens diameter is 10 um. The target phase map, shown in Figure S8a, was obtained by matching the simulated phase response to the orientation angles of the meta-atoms. The spatial rotation of the meta-atoms was subsequently optimized to reproduce the desired phase distribution, resulting in the phase profile presented in Figure S8b and enabling accurate wavefront manipulation.

Based on the reciprocity principle, the far-field emission of a gain medium surrounding the nanolaser BIC metalens was modeled in an equivalent manner to external optical illumination.[2] Three-dimensional finite-difference time-domain (FDTD) simulations were performed using Lumerical, with circularly polarized incident light as the excitation source. A two-dimensional transmission monitor positioned in the x–z plane was employed to evaluate the focusing characteristics of the nanolaser metalens, as illustrated in Figure S8c. The simulation results demonstrated diffraction-limited focusing performance, as shown in Figure S8d. Nevertheless, small side lobes were present in the simulated focal spot, which can be attributed to residual phase errors and scattering effects.

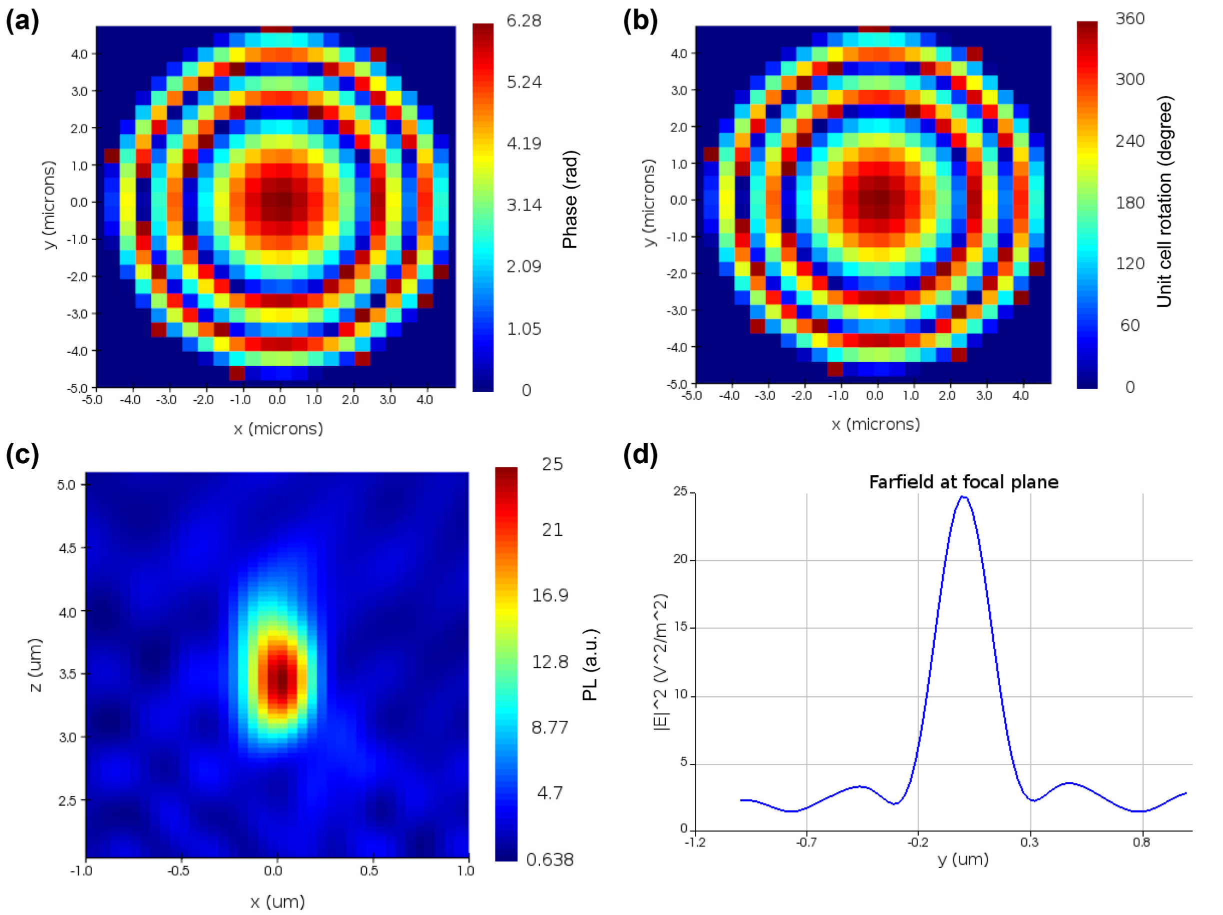

Figure S8. Nanolaser metalens design. (a) Target phase map of the focusing nanolaser metalens. (b) Reconstructed phase map obtained through unit-cell rotation. (c) Simulated far-field focusing distribution of the nanolaser metalens in the *x*–*z* plane. (d) Simulated focal intensity distribution of the nanolaser metalens at the focal plane.

S10. PINNs nanolaser beam steering

The beam-steering nanolaser was designed by engineering a linear phase gradient across the metasurface to redirect the emitted beam toward a predetermined angle while preserving the high directional emission of the BIC nanolaser. Unlike the focusing metalens, which employs a hyperbolic phase profile, the beam-steering device utilizes a linear phase distribution described by:[1]

$$\phi(x, y) = \frac{2\pi}{\lambda} x \sin(\theta_x) \tag{S19}$$

where ($\boldsymbol{\lambda}$ = 620 nm) is the operating wavelength, and θ_x denote the steering angles along the *x*-directions, respectively. The target phase map, shown in Figure S9a, was generated by mapping the required phase distribution to the corresponding meta-atom rotation angles. The spatial orientation of the meta-atoms was subsequently optimized to accurately reproduce the desired linear phase gradient, resulting in the phase profile presented in Figure S9b and enabling efficient beam steering. 3D FDTD simulations were performed in Lumerical using circularly polarized incident light. A two-dimensional transmission monitor located in the *x-z* plane was employed to characterize the far-field steering performance, as illustrated in Figure S9c. The simulation results confirm that the emitted beam is redirected toward the designed steering angle with a well-defined far-field profile, demonstrating effective wavefront control through the engineered metasurface phase gradient.

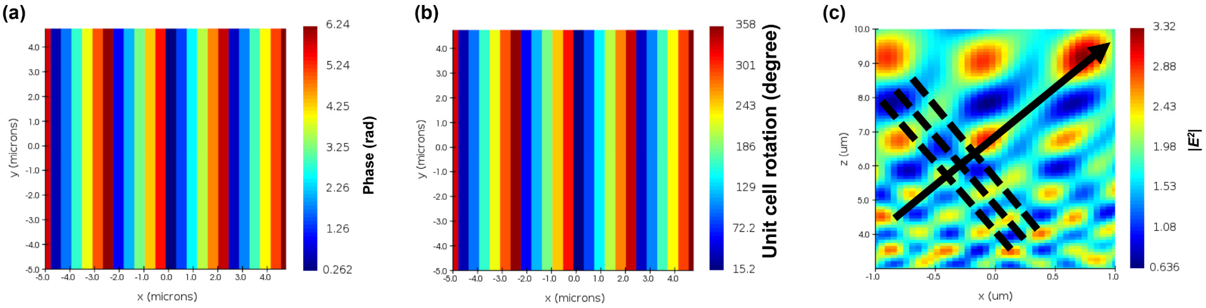

Figure S9. Nanolaser beam-steering design. (a) Target phase map of the beam-steering nanolaser. (b) Reconstructed phase map obtained through unit-cell rotation. (c) Simulated far-field beam steering of the nanolaser in the *x-z* plane.

S11. Benchmark of NanoPhotoNet-Lase with AI and PINN Design Models

Recent advances in AI have significantly accelerated the design of metasurfaces by replacing computationally intensive electromagnetic simulations with surrogate neural network models. Most existing approaches, however, have focused on passive dielectric metasurfaces and optical scattering problems, where the objective is to predict reflection, transmission, or phase responses from geometric parameters. Standard PINN have recently demonstrated excellent capability for solving Maxwell's equations and accelerating inverse electromagnetic design of passive structures, while complex-valued neural networks (CVNNs) have been introduced to better represent complex electromagnetic fields in reconfigurable metasurfaces.[3] Despite these advances, existing AI frameworks generally do not incorporate the physical processes governing optical gain, stimulated emission, carrier population dynamics, or lasing thresholds, making them unsuitable for the inverse design of active metasurface nanolasers.

Table S1 compares NanoPhotoNet-Lase with representative AI-based inverse design frameworks reported in the recent literature. Unlike previous PINN models that solve only Maxwell's equations for passive electromagnetic scattering,[4] NanoPhotoNet-Lase simultaneously embeds the vector Helmholtz equation together with the four-level laser rate equations into a unified physics-informed optimization framework. This coupled formulation enables self-consistent prediction of both the electromagnetic cavity response and gain dynamics, allowing the network to directly estimate lasing characteristics from arbitrary metasurface geometries without relying solely on labeled simulation datasets.

Compared with conventional PINNs, NanoPhotoNet-Lase extends the application of physics-informed learning from passive optical scattering to active light-emitting metasurfaces supporting quasi-BICs. The model is specifically developed for Rd B gain-assisted Nb_2O_5 metasurface nanolasers operating at 620 nm, where the high-Q quasi-BIC resonance provides strong optical confinement required for low-threshold coherent emission. As summarized in Table S1, NanoPhotoNet-Lase predicts a Q-factor of 264 while simultaneously optimizing photoluminescence amplification, lasing performance, and resonant cavity geometry within a single inverse-design framework. In contrast, previously reported PINN implementations are restricted to passive wave propagation or fabrication-aware electromagnetic modeling and do not predict gain dynamics, photoluminescence amplification, or lasing characteristics.[5]

Another important distinction is the multifunctionality enabled by the proposed framework. Existing AI approaches generally optimize either optical phase profiles or resonant spectra independently. NanoPhotoNet-Lase simultaneously performs inverse design of active metasurface resonances together with phase-gradient functionalities, enabling coherent phase-gradient metalenses and beam-steering nanolaser arrays using the same physics-informed model. Consequently, the framework supports both wavefront engineering and coherent light generation, whereas previous PINN and CVNN models primarily target passive beam manipulation, resonance prediction, or reconfigurable absorbers.

The benchmark also highlights the broader design capability of NanoPhotoNet-Lase. Besides optimizing a single lasing wavelength at 620 nm, the framework predicts a photoluminescence dimensional tuning range from 620 to 695 nm through metasurface geometry optimization, providing a substantially larger active tuning space than previous AI models that do not support wavelength-tunable photoluminescence or active gain optimization. Furthermore, NanoPhotoNet-Lase directly incorporates the fluorescent gain medium into the learning process, allowing

simultaneous optimization of cavity resonance, gain overlap, and lasing performance, capabilities absent from existing passive AI frameworks.

Overall, NanoPhotoNet-Lase represents a significant extension of physics-informed deep learning from passive electromagnetic inverse design toward physically consistent active nanophotonic systems. By embedding both Maxwell's equations and laser rate equations into a unified optimization framework, the model provides a computationally efficient, scalable, and physically interpretable approach for the inverse design of coherent metasurface nanolasers, programmable phase-gradient metalenses, and beam-steering active photonic devices. This capability substantially broadens the application scope of physics-informed AI beyond passive metasurface design and establishes a general methodology for future AI-assisted active nanophotonic systems.

Table S1. Comparison of Data-Driven and Physics Informed Inverse Design Models for Active Metasurfaces, Tunable Resonances, and Amplified PL.

**Parameter**	**Our work**	**Standard PINN [4]**	**Standard PINN** [5]	**CVNN for Phase-Change [3]**
**Model Architecture**	**NanoPhotoNet-Lase**	Standard PINN	Standard PINN	Complex-Valued Neural Network
**Physical Constraints**	Maxwell's + Four-Level Rate Equations	Maxwell's Equations	Maxwell's Equations	None (Data-driven)
**Metasurface Resonance**	Quasi-BICs	N/A	N/A	Multiple Resonances
**Operating Wavelength (nm)**	620	740~750	632.8	2000~4000
**Fluorescent/Gain Medium**	Rhodamine B	N/A (Passive)	N/A (Passive)	N/A (Passive)
**Predicted Q-Factor**	**264**	N/A	N/A	80~100
**PL Amplification Factor**	264	N/A	N/A	N/A
**PL Dimensional Tuning Range (nm)**	620~695	N/A	No	3000~4000
**Phase Gradient (Metalens)**	Yes	Yes (phase only)	No	No
**Phase Gradient (Beam steering)**	Yes	Yes (phase only)	No	No
**Application Scope**	Inverse design lasing, PL focusing and, beam steering	Passive wave scattering	Inverse design with fabrication variations	Reconfigurable absorbers

References

[1] M. Khorasaninejad, W. T. Chen, R. C. Devlin, J. Oh, A. Y. Zhu, and F. Capasso, "Metalenses at visible wavelengths: Diffraction-limited focusing and subwavelength resolution imaging," *Science,* vol. 352, no. 6290, pp. 1190-1194, 2016.

[2] A. Vaskin, R. Kolkowski, A. F. Koenderink, and I. Staude, "Light-emitting metasurfaces," *Nanophotonics,* vol. 8, no. 7, pp. 1151-1198, 2019.

[3] I. Tanriover, W. Hadibrata, J. Scheuer, and K. Aydin, "Neural networks enabled forward and inverse design of reconfigurable metasurfaces," *Optics Express,* vol. 29, no. 17, pp. 27219-27227, 2021/08/16 2021, doi: 10.1364/OE.430704.

[4] V. Medvedev, A. Erdmann, and A. Rosskopf, "Physics-informed deep learning for 3D modeling of light diffraction from optical metasurfaces," *Optics Express,* vol. 33, no. 1, pp. 1371-1384, 2025/01/13 2025, doi: 10.1364/OE.544116.

[5] J. Chen *et al.*, "Neural Network-Based Inverse Design of Metasurfaces with a Physics-Informed Intelligent Algorithm," in *2025 International Applied Computational Electromagnetics Society Symposium (ACES-China)*, 8-11 Aug. 2025 2025, pp. 1-3, doi: 10.23919/ACES-China66523.2025.11332992.
